# Real world evidence of dupilumab effectiveness in a Colombian cohort of patients diagnosed with severe asthma

**DOI:** 10.3389/falgy.2025.1564033

**Published:** 2025-05-13

**Authors:** Abraham Alí-Munive, Josefina Zakzuk, Nelson J. Alvis-Zakzuk, Elizabeth García, Claudia Diaz Bossa, Diana Jimena Cano Rosales, Fabio Bolívar, Alejandro Carreño, Paula Rodríguez-Ordoñez, Natalia Gómez-Ardila, Gabriel Patiño, Sergio Londoño, Carlos A. Torres-Duque

**Affiliations:** ^1^ Asmaire ReXpira Program, Fundación Neumológica Colombiana, Bogotá, Colombia; ^2^ Institute for Immunological Research, University of Cartagena, Cartagena, Bolívar, Colombia; ^3^ ALZAK Research Group, ALZAK, Cartagena, Bolívar, Colombia; ^4^ Department of Health Sciences, Universidad de la Costa, Barranquilla, Colombia; ^5^ Allergy Unit, UNIMEQ-ORL, Bogotá, Colombia; ^6^ School of Medicine, Universidad de Los Andes, Bogotá, Colombia; ^7^ Research Department, NEUMOMED, Medellín, Antioquia, Colombia; ^8^ Asthma Program, Instituto Neumológico del Oriente, Bucaramanga, Colombia; ^9^ Department of Internal Medicine, Universidad Autónoma de Bucaramanga, Bucaramanga, Santander, Colombia; ^10^ Research Department, Centro de Alergología Alejandro Carreño, Barranquilla, Atlántico, Colombia; ^11^ Health Economics and Value Assessment, Sanofi, Bogotá, Colombia; ^12^ Medical Area, Sanofi, Bogotá, Colombia; ^13^ Market Access, Sanofi, Bogotá, Colombia

**Keywords:** asthma, dupilumab, treatment outcome, Colombia, effectiveness

## Abstract

**Background:**

Real-world effectiveness and safety of dupilumab for asthma treatment have been evaluated in USA and Europe, but research from Latin America is lacking. We aimed to describe the effectiveness of dupilumab in terms of changes in the annual rate of asthma exacerbations (AER) and their impact on lung function in Colombian patients.

**Methods:**

Real-world, descriptive, and multi-centric (five clinical centers located in four different cities in Colombia) retrospective study that included patients aged ≥18 years with severe asthma, as defined by the GINA criteria. Data were collected from medical records of medical centers specialized in pulmonology or allergy care) spanning from 12 months before the prescription of dupilumab (baseline) to 25 months later. Follow-up data were categorized at various time points (2–4, 5–7, 8–10, 11–13, 14–18, and 19–25 months). Main outcomes were annual rates of asthma exacerbations (emergency visits or hospitalizations due to asthma), lung function measured through FEV_1_ and percent predicted FEV_1_ (FEV_1_pp), and Asthma Control Test (ACT) scores. Outcome rates were compared between baseline and follow-up data points. FeNO and absolute eosinophil counts throughout the observed period was also explored.

**Results:**

A total of 98 patients were included. At baseline, the mean AER was 0.61 ± 1.45 per adult. Lower AER were observed after one (0.11 ± 0.54) or two-years (0.08 ± 0.20) of dupilumab treatment (*p* = 0.03). FEV1 measurements after one or two years of dupilumab treatment were significantly lower than baseline (*p* = 0.03). Mean change from baseline in FEV_1_ was 302.1 ± 481.97 ml (*n* = 19), 282.00 ± 231.99 ml (*n* = 10), and 248.18 ± 281.21 ml (*n* = 11) in the 2–4-, 11–13-, and 19–25-month follow-up periods, respectively. FEV_1_pp showed higher but not significant values from the 2–4-month period, with a median change of 12.5% (IQR: 0.3, 21.5). The proportion of patients with uncontrolled asthma (ACT ≤15) decreased from 68% at baseline to 19% and 20% at year-one and second year of treatment, respectively (*p* = 0.003). The proportion of patients reaching FeNO values below 25 ppb was lower after dupilumab treatment than in baseline (*p* < 0.0001). Of the total cohort (*n* = 99), 15 (15.2%) experienced an adverse event (AE). Three patients discontinued dupilumab permanently, and two discontinued dupilumab due to AEs.

**Conclusions:**

Dupilumab is an effective and well-tolerated treatment for severe asthma in Colombia, resulting in reduced exacerbations and improved asthma control, lung function, and FeNO levels.

## 1 Introduction

Asthma remains one of the most prevalent chronic respiratory diseases worldwide, with a significant public health challenge, and persisting as a life-threatening condition (1, 2). While most individuals with asthma experience manageable symptoms, a subset, comprising approximately 5%–10%, faces severe manifestations, requiring high doses of inhaled corticosteroids (ICS) and long-acting beta-agonist (LABA), and episodically oral corticosteroids (OCS) (3, 4). This subset is classified as severe asthma (SA). SA is associated with a lower quality of life, greater risk of severe exacerbations and mortality, and increased healthcare costs (5, 6). According to the European Respiratory Society and the American Thoracic Society guidelines, SA is defined as “asthma which requires treatment with high-dose inhaled corticosteroids (ICS) plus a second controller medication and/or systemic corticosteroids to prevent it from becoming “uncontrolled” or which remains “uncontrolled” despite this therapy” (7). Conversely, GINA defines SA as a condition requiring high-intensity treatment to maintain good control, or a lack of good control despite high-intensity treatment (4).

Dupilumab, an antagonist of the alpha-chain interleukin (IL)-4 receptor, disrupts the IL-4 and IL-13 signaling pathways, key drivers of T helper 2 (Th2) inflammation observed in various allergic diseases, including asthma (8). The efficacy and safety of dupilumab for asthma treatment have been extensively evaluated in phase III randomized clinical trials (RCT) and subsequent analyses, yielding satisfactory results (9). This monoclonal antibody intervention significantly reduced asthma exacerbations and improved prebronchodilator forced expiratory volume in the first second (FEV1) in patients with uncontrolled moderate-to-severe asthma (9–12). However, there remains a gap in our understanding of the real-world (RW) effectiveness of dupilumab, particularly in populations not represented in RCTs, such as the Colombian population. RCTs provide robust evidence about the efficacy and safety of a treatment under ideal conditions, but they may not fully reflect responses in diverse populations. RW observational studies are crucial for providing complementary evidence on the practical efficacy and safety of interventions, addressing potential discrepancies between trial populations and RW patients. Most studies evaluating the effectiveness of dupilumab as a treatment option for SA have been done in Europe, US, and Japan (13–15). No studies in Latin-American populations have been published hitherto. In Colombia, where dupilumab has been utilized as an add-on therapy for SA since its approval in 2019, there is a lack of published data on its clinical effectiveness and local experience. Documenting and analyzing local experiences, has the potential to improve clinical practices and treatment guidelines for SA in Colombia and contribute to a more personalized and contextualized approach in the management of this chronic disease globally. Therefore, this study aimed to describe changes in asthma exacerbation, lung function, T2 inflammation biomarkers and other outcomes during a specific follow-up period in patients with SA treated with dupilumab in RW settings in Colombia.

## 2 Methods

### 2.1 Study design and population

This was a RW, descriptive, and multi-centric retrospective study involving five clinical centers located in four different cities in Colombia: Fundación Neumológica Colombiana and UNIMEQ ORL (Bogotá), Neumomed IPS (Medellín), Instituto Neumológico del Oriente (Bucaramanga), and Centro de Alergología Alejandro Carreño (Barranquilla). These medical centers are part of the private sector and receive mostly patients affiliated to the public health system of Colombia. These urban centers, representing different regions of the country (Andean, Pacific, and Caribbean), are characterized by diverse populations in terms of ethnicity and socioeconomic status. The majority of the population in these areas is covered by the national public health insurance system.

Eligible patients were identified from medical records at participating institutions between April 2019 and May 2023. We included patients aged 18 years or older receiving dupilumab, with a documented diagnosis of asthma (ICD-10: J45 or J46) in the medical record at least one year before the prescription of the biologic, receiving dupilumab therapy, and classified as having SA according to the GINA guidelines at least 1–2 months prior to initiating dupilumab (4).

Patient selection followed these criteria: (1) documentation of severe asthma and prescription of dupilumab; (2) confirmation of at least one additional medical control visit after the index date; (3) a final clinical control recorded at least six months after the index date; and (4) verification of a minimum of one year of baseline observation with a persistent diagnosis of asthma during that period.

We excluded patients with less than 6 months of observation after dupilumab prescription and/or with a co-diagnosis of chronic obstructive pulmonary disease (COPD) or other chronic respiratory diseases, such as bronchiectasis or tuberculosis, reported in the medical record at any time during the one-year observation period before dupilumab prescription. If, during the observation window, any center reported a patient who had discontinued dupilumab before completing six months of therapy for reasons unrelated to treatment failure, information on that case was retained for safety reporting, but clinical endpoints related to asthma control were not assessed.

### 2.2 Data collection and follow up definition

An electronic case report form (eCRF), developed in KoboToolbox, was used for data collection from medical records. Personal data were accessed once by designated research members; however, no data allowing patient identification were registered in the eCRF. Data consistency checks, outlier identification, and missing data handling were performed before analysis.

The day of dupilumab prescription was considered as the index date. This prescription date also represents the first time the patient receives the biologic. The baseline characteristics were assessed considering the 12-month period preceding the index date. All medical visits during dupilumab treatment were recorded with the same structured form. Clinical variables included: asthma control, lung function, healthcare resources utilization (HCRU) and medication prescriptions. Participating investigator physicians were invited to enter into the eCRF all clinical visits occurring after the initiation of the biologic therapy up to the latest permitted date. All recorded visits followed the same structured data capture format for entering outcomes and other reported variables of interest.

The follow-up period began on the index date and continued through the last available medical visit recorded for each patient. Follow-up duration varied across patients; however, the earliest possible date for entering the observation period was April 1, 2019, and the latest possible date for follow-up data was May 31, 2023.

### 2.3 Baseline characteristics

Demographic data collected at baseline included age, sex, socioeconomic stratum (Colombian classification from 1 to 6 based on dwelling characteristics and its surroundings), height, weight, department, working status, patient affiliation regime to social security (contributive/subsidized/special), educational level, and occupational status. The clinical variables analyzed included age at disease onset, comorbidities, and baseline pharmacotherapy, including biologic agents and immunotherapy. Asthma control metrics were evaluated using the Asthma Control Questionnaire-5 (ACQ-5) and Asthma Control Test (ACT). Quality of life was assessed using the Asthma Quality of Life Questionnaire (AQLQ). Other parameters included blood eosinophil count (BEC), fractional exhaled nitric oxide (FeNO) levels, pre-bronchodilator forced expiratory volume in one second (FEV1) values, and total immunoglobulin E (IgE) concentrations. Comorbidities were identified using ICD-10 codes documented in the medical records.

### 2.4 Outcomes

The main outcomes were asthma exacerbations, defined as an emergency room visit or hospitalization due to exacerbation, and lung function measured through FEV_1_ and the percent predicted FEV_1_ (FEV_1pp_).

The number of emergency room visits, and hospitalization and intensive care unit admissions due to asthma exacerbations are described as an information source for health care resource use (HCRU). Oral and inhaled corticosteroid use (dosage, frequency, and duration) were also described during the study period. Additionally, the study examined changes in asthma control using ACT scores. Presentation of common adverse events associated with dupilumab usage was reported.

Values of FeNO, total IgE values, BEC, and clinical asthma remission were explored when data were available. For FeNO, values below 25 ppb were interpreted as negative for airway inflammation. Clinical asthma remission was defined using three criteria for at least one year or in two follow-up time points separated by at least one year: 1) absence of exacerbations requiring oral corticosteroids or hospitalization, 2) an ACQ score ≤1.5 or an ACT score ≥20, and 3) an FEV_1_pp ≥80% or an improvement in FEV_1_ ≥ 100 ml.

### 2.5 Statistical analysis

Categorical variables were presented using absolute and relative frequency distributions. Numerical variables with a normal distribution were described using the mean and standard deviation (SD), while those not following a normal distribution were characterized using the median and interquartile range (IQR; first quartile—third quartile). Normality of the data was assessed using the Kolmogorov–Smirnov test.

Changes from baseline in lung function parameters, ACT and biologic markers (BEC and FeNO) were described at different follow-up time points (2–4, 5–7, 8–10, 11–13, 14–18, and 19–25 months) according to data availability for each patient. Due to structural characteristics of the Colombian healthcare system, delays in scheduling follow-up visits are common. Therefore, clinical assessments were grouped into standardized time-points (3, 6, 9, and 12 months), allowing a ± 1 month margin around each interval (e.g., the 3-month follow-up window included visits conducted between 2 and 4 months after treatment initiation). Post–12-month follow-up data were categorized into two descriptive intervals: 14–18 months and 19–25 months. In cases where temporary discontinuation of dupilumab longer than two months was detected, the analysis window was restricted to the date of the last prescription prior to interruption.

To obtain representative information on asthma control for each treatment year, we identified a single follow-up visit per patient corresponding to the end of the first year (T1 = 11–13 months) and the second year (T2 = 18–24 months) of treatment. These time points were selected to summarize the cumulative impact of dupilumab over each annual period. AER and mean FEV_1_ and FEV_1 pp_ parameters were assessed at baseline, T1 and T2. AER represents the cumulative number of ER or hospitalization events recorded in each medical control during the first and second year of follow-up. Annualized rates were calculated as a ratio between the number of events and the number of months of follow-up and multiplied by 12. Comparisons of continuous variables across the three time points (baseline, T1, and T2) were performed using the Kruskal–Wallis test, due to the non-parametric distribution of the data. For categorical variables, such as the proportion of patients experiencing exacerbations, the chi-squared test was used. All statistical tests were two-sided, and a *p*-value < 0.05 was considered statistically significant. Statistical analyses were performed using R software (version 4.4.0 “Angel Food Cake”; R Foundation for Statistical Computing, Vienna, Austria).

## 3 Results

### 3.1 Baseline clinical and sociodemographic characteristics

Ninety-eight SA cases met the eligibility criteria for this study, including 6 or more months of using dupilumab. Table 1 shows descriptive sociodemographic features of the included cohort is (*n* = 98). The mean body mass index was 27.7 ± 5.0; 34 (39.5%) were overweight, and 23 (26.7%) were obese. The most common comorbidities were rhinosinusitis with nasal polyps (62.2%, *n* = 61) and allergic rhinitis (41.8%, *n* = 41). OCS were prescribed for disease control in 23.5% (*n* = 23) of patients. The mean treatment duration with dupilumab was 14.4 ± 7.1 months (Supplementary Table S1).

**Table 1:** Sociodemographic characteristics of the cohort (*N* = 98).

Variables^1^	Value^2^
Age, years	
Mean (SD)	47.5 (13.5)
Sex	
Women	73 (74.5%)
Men	25 (25.5%)
BMI (*n* = 86)	
Mean (SD)	27.7 (5.0)
Department	
Antioquia	25 (25.5%)
Atlantico	4 (4.1%)
Bogota, D.C.	48 (49.0%)
Cundinamarca	7 (7.1%)
Norte De Santander	1 (1.0%)
Santander	12 (12.2%)
Tolima	1 (1.0%)
Socioeconomical stratum	
1–2	16 (16.3%)
3–4	20 (20.4%)
5	2 (2.0%)
No reported	60 (61.2%)
Educational level	
Elementary school	3 (3.1%)
High school	17 (17.3%)
Technician/college	39 (39.8%)
Postgraduate	3 (3.1%)
No reported	36 (36.7%)
Current occupation	
Worker	64 (65.3%)
Student	4 (4.1%)
Retired	12 (12.2%)
Homemaker	15 (15.3%)
Other	2 (2.0%)
Unemployed	1 (1.0%)
Clinical care center	
Fundación neumológica Colombiana	30 (30.6%)
UNIMEQ ORL	28 (28.6%)
Neumomed IPS	25 (25.5%)
Instituto Neumológico del Oriente	11 (11.2%)
Centro de Alergología Alejandro Carreño	4 (4.1%)
Patient affiliation regime to social security	
Contributive	89 (90.8%)
Subsidized	4 (4.1%)
Special	1 (1.0%)
No reported	4 (4.1%)

^1^ Since for some variables, data was not retrieved for the medical record in all patients, the total number of patients (*N*=) is reported among parentheses.

^2^ *n* (%); Mean (SD). Socioeconomic stratification in Colombia is a composite index based on housing type, overcrowding, access to basic services, income, and educational attainment, as defined by national legislation (Law 142 of 1,994). The index ranges from 1 to 6, with 1 representing the most vulnerable households.

The clinical characteristics of patients are presented in Table 2. Twenty-four patients (24.5%) experienced at least one episode of severe exacerbation in the last year (Table 2); among this subgroup, the average number of exacerbations was 2.5 ± SD 1.98. Most patients (69.3%, *n* = 52) had uncontrolled asthma (ACT ≤15). Furthermore, pulmonary function tests indicate a mean FEV_1_ of 1,782.5 ± 740.6 ml and a median FeNO of 47.0 (IQR: 20.5, 96.0) ppb, while IgE levels median of 183.5 (IQR: 52.0, 685.0) IU/ml and BEC of 350.0 (IQR: 165.0, 635.0) cells/µl.

**Table 2:** Baseline clinical characteristics.

Variables^1^	Value^2^
Age of disease onset (*N* = 73)	
Mean (SD)	28.45 (16.27)
Comorbidities (*N* = 98)	
Rhinosinusitis with nasal polyps	61 (62.2%)
Allergic rhinitis	41 (41.8%)
Intolerance to NSAIDs	35 (35.7%)
Gastroesophageal reflux	22 (22.4%)
Drug allergy	18 (18.4%)
Atopic dermatitis	13 (13.3%)
Hypertension	10 (10.2%)
Conjunctivitis	8 (8.2%)
Sinusitis	8 (8.2%)
Food allergy	7 (7.1%)
Depression	6 (6.1%)
Urticaria	6 (6.1%)
Anxiety	4 (4.1%)
Coronary heart disease	1 (1.0%)
Heart failure	1 (1.0%)
Other comorbidities	37 (37.8%)
Pharmacological therapy (*N* = 98)	
ICS	10 (10.2%)
ICS/LABA	97 (99.0%)
LAMA	61 (62.2%)
Leukotriene modifiers	73 (74.5%)
SABA	73 (74.5%)
Biological drugs	24 (24.5%)
Ipratropium bromide	5 (5.1%)
Oral corticosteroid	23 (23.5%)
Immunotherapy	11 (11.2%)
Theophylline	1 (1.0%)
Biologic drugs before dupilumab initiation (*N* = 24)	
Benralizumab	7 (29.2%)
Mepolizumab	6 (25.0%)
Omalizumab	11 (45.8%)
Immunotherapy (*N* = 11)	
Grasses	1 (9.1%)
HDM	9 (81.8%)
Other	2 (18.2%)
Dupilumab dosage (*N* = 98)	
400/200 mg	43 (43.9%)
600/300 mg	55 (56.1%)
Treatment duration, months	
Mean (SD)	14.39 (7.08)
Disease control (*N* = 98)	
Severe exacerbation	
No	74 (75.5%)
Yes	24 (24.5%)
ACT score (*N* = 75)	
Median (IQR)	13.0 (11.0, 17.0)
ACT score ranges (*n* = 75)	
≥20	11 (14.7%)
16–19	12 (16.0%)
≤15	52 (69.3%)
ACQ5 score (*N* = 48)	
Median (IQR)	2.20 (1.50, 3.20)
ACQ5 score ranges (*N* = 48)	
<0.75	3 (6.2%)
0.75–1.50	10 (20.8%)
>1.50	35 (72.9%)
FEV_1_ (*n* = 67)	
Mean (SD)	1,782.5 (740.6)
FEV_1pp_ (*N* = 73)	
Mean (SD)	67.36 (26.0)
FeNO (*n* = 42)	
Median (IQR)	47.0 (20.5, 96.0)
IgE (*n* = 54)	
Median (IQR)	183.5 (52.0, 685.0)
Eosinophils (*N* = 63)	
Median (IQR)	350.0 (165.0, 635.0)

^1^ Since for some variables, data was not retrieved for the medical record in all patients, the total number of patients (*N*=) is reported among parentheses.

^2^ *n* (%); Mean (SD); Median (IQR). ICS, Inhaled corticosteroids; LABA, Long-acting beta-agonist; LAMA, Long-acting muscarinic antagonist; SABA, Short-acting beta-agonist; HDM, House dust, mite; ACT, Asthma control test; ACQ-5, Asthma Control Questionnaire—5; FEV_1_, Forced expiratory volume in one second; FEV_1pp_, Forced Expiratory Volume in one second percent predicted; FeNO, Fractional exhaled nitric oxide; IgE, Immunoglobulin E.

### 3.2 Exacerbations

**Figure 1:**
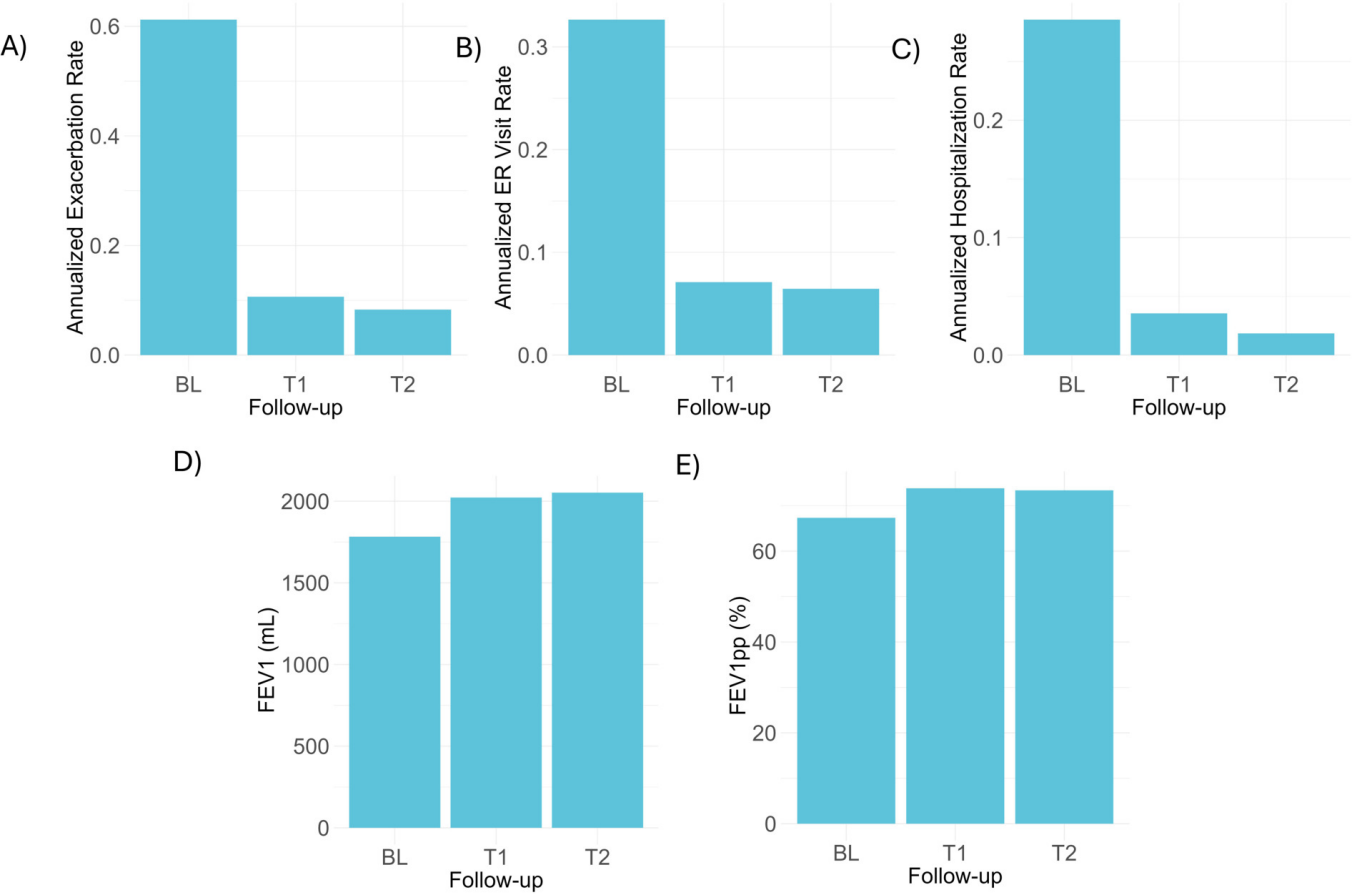
Frequency of exacerbations and lung function before and after dupilumab treatment. Annualized exacerbation **(A)**, emergency room visit **(B)**, and hospitalization rates **(C)** at baseline and during the first (T1) or second year (T2) of treatment with dupilumab. Mean FEV_1_ and FEV_1pp_ values are also presented before and after treatment with the biologic. BL, baseline; ER, emergency room; FEV_1_, forced expiratory volume in one second, FEV_1pp_, forced expiratory volume in one second percent predicted.

The AERs were significantly lower after dupilumab treatment compared to baseline (*p* = 0.03). Compared to baseline AER (0.61 ± 1.45 per adult), a reduction of 82.0% and 86.9% in T1 and T2 AERs were observed, respectively (Figure 1 and Supplementary Table S2). ICU admissions were registered for five patients (5.1%) at baseline. After dupilumab, only one ICU admission occurred within the first year of initiating.

### 3.3 Lung function

**Figure 2:**
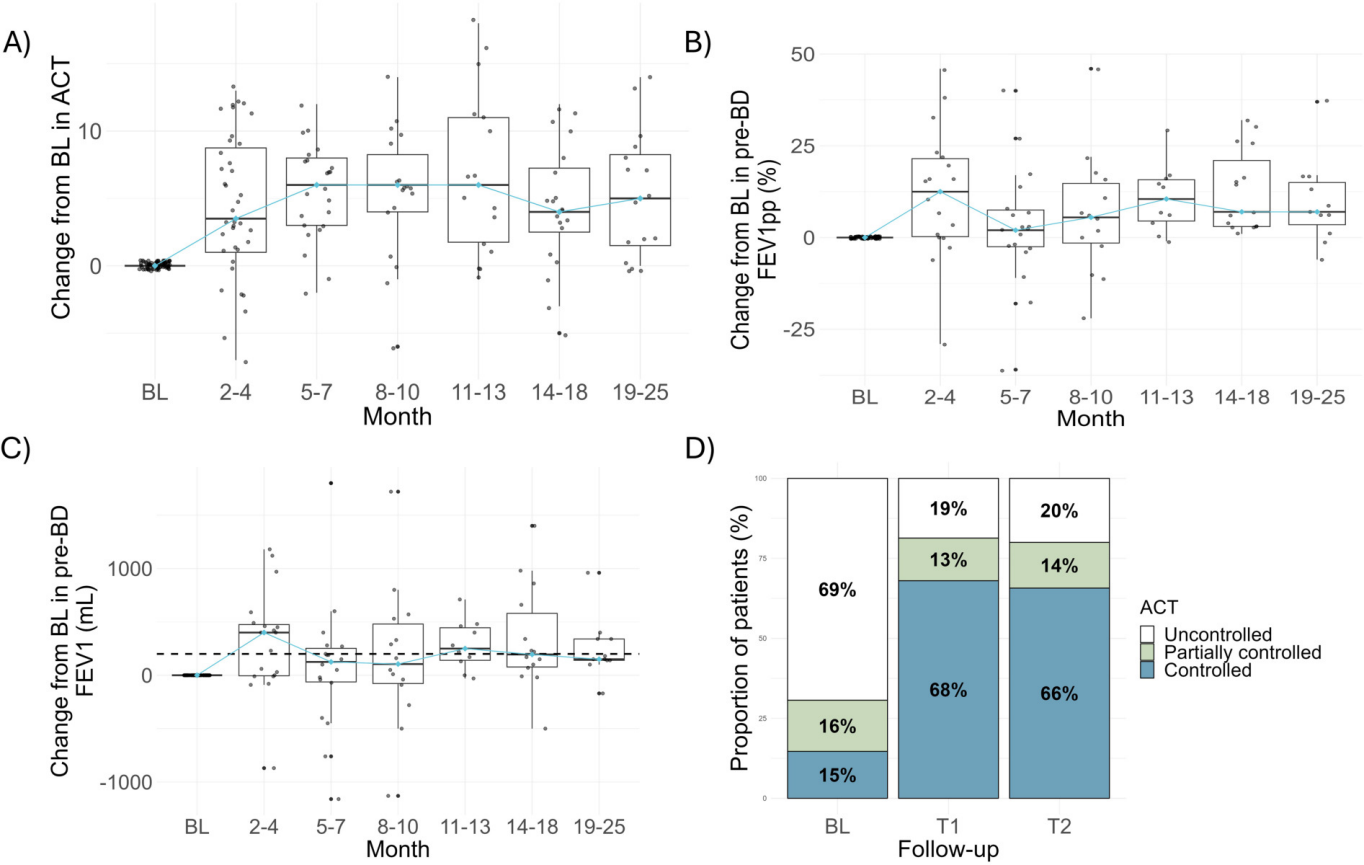
Changes from baseline in lung function and asthma control during two years of treatment with dupilumab. Changes in baseline in FEV_1_ **(A)** and FEV_1pp_ **(B)** before bronchodilator at different time-points during dupilumab treatment are shown. **(C)** Changes in the Asthma Control Test are presented. **(D)** Based on ACT, relative frequencies of controlled, partially controlled and uncontrolled asthma were calculated at baseline, one (T1) or second year (T2) of treatment. Dashed line in A was set at 200 mL to highlight patients with improvements over this value. Blue line in all box plots is connecting median values. BL, baseline; FEV_1_, forced expiratory volume in one second; FEV_1pp_, forced expiratory volume in one second percent predicted.

As shown in Figure 1, mean FEV_1_ values were higher at one-year (2,022.5 ± 766.4 ml) and second year (2,052.3 ± 759.6 ml) of follow-up after treatment with dupilumab, compared to baseline (1,782.5 ± 740.5 ml) reaching statistical significance (*p* = 0.03). Higher FEV_1_ values were observed from the 2–4-month time-point and median changes were greater than 200 ml in most follow-up controls (Table 3 and Figure 2). Mean FEV_1pp_ was 67.4% at baseline, and 73.9% at T1 and 73.4% at T2 (*p* = 0.14). Changes in FEV_1pp_ showed improvement from the 2 to 4-month period, with a median change of 12.5% (IQR: 0.3, 21.5) (Table 3).

**Table 3:** Measured values and changes from baseline in FEV1 and percent predicted FEV1 (FEV1pp) at baseline and follow-up time point.

	BL	2–4	5–7	8–10	11–13	14–18	19–25
Absolute values							
FEV_1_ (ml)							
N	67	22	23	15	12	16	11
Mean (SD)	1,782.54 (740.56)	1,832.73 (538.23)	2,069.13 (948.43)	2,142.00 (646.61)	1,861.67 (646.39)	1,856.25 (792.03)	2,373.64 (584.90)
Median (IQR)	1,720.00 (1,210.00, 2,400.00)	1,820.00 (1,590.00, 2,165.00)	1,870.00 (1,460.00, 2,475.00)	2,210.00 (1,565.00, 2,560.00)	1,840.00 (1,545.00, 1,985.00)	1,800.00 (1,345.00, 2,315.00)	2,290.00 (1,905.00, 2,700.00)
FEV_1pp_							
N	73	21	23	16	12	16	12
Mean (SD)	67.36 (25.97)	70.43 (19.75)	69.65 (24.38)	80.81 (23.53)	65.92 (23.73)	68.00 (25.86)	83.00 (23.71)
Median (IQR)	62.00 (45.00, 89.00)	69.00 (59.00, 87.00)	75.00 (51.50, 88.50)	79.50 (63.25, 101.00)	63.00 (51.25, 76.25)	67.50 (56.50, 79.50)	82.00 (61.50, 101.75)
Change from baseline							
FEV_1_ (ml)							
N	67	19	18	14	10	14	11
Mean (SD)	0.00 (0.00)	302.11 (481.97)	73.89 (606.48)	172.86 (657.62)	282.00 (231.99)	332.14 (487.13)	248.18 (281.21)
Median (IQR)	0.00 (0.00, 0.00)	400.00 (−5.00, 475.00)	125.00 (−62.50, 252.50)	105.00 (−77.50, 480.00)	250.00 (140.00, 445.00)	195.00 (77.50, 580.00)	150.00 (140.00, 340.00)
FEV_1pp_ (%)							
N	73	18	19	14	10	15	11
Mean (SD)	0.00 (0.00)	11.78 (17.74)	2.84 (16.00)	6.29 (16.57)	10.70 (9.21)	12.60 (11.05)	9.64 (11.55)
Median (IQR)	0.00 (0.00, 0.00)	12.50 (0.25, 21.50)	2.00 (−2.50, 7.50)	5.50 (−1.50, 14.75)	10.50 (4.50, 15.75)	7.00 (3.00, 21.00)	7.00 (3.50, 15.00)

FEV_1_, Forced expiratory volume in one second; FEV_1pp_, Forced Expiratory Volume in one second percent predicted.

### 3.4 Oral corticosteroid prescriptions

OCS prescriptions decreased from 20.4% (*n* = 20 out of 98 patients) at baseline to 2.5% (*n* = 1 out of 40 patients) at the 8–10 months period (Supplementary Table S3). In subsequent periods, while the percentages fluctuate, they consistently remain lower than those reported at baseline. The use of ICS/LABA medications remained unchanged throughout the analyzed periods, with minimal meaningful changes (Supplementary Table S3).

### 3.5 Asthma control

At baseline, 68% of patients had uncontrolled asthma. After dupilumab, lower rates of uncontrolled asthma were observed at year-one (19%) and second year of treatment (20%), respectively (chi^2 =^ 47.14^,^ *p* < 0.0001). Positive changes in ACT values were observed from 2 to 4 months (Supplementary Table S4 and Figure 2).

### 3.6 Feno

**Figure 3:**
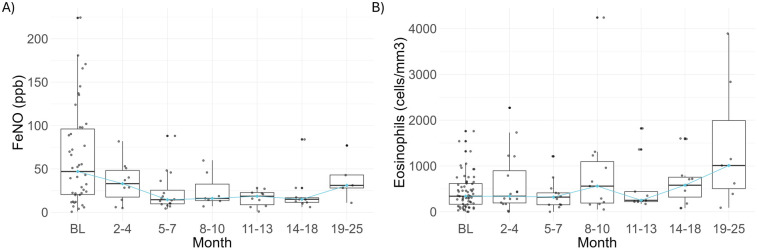
Assessment of type 2 inflammation markers during dupilumab treatment. **(A)** FeNO and **(B)** absolute eosinophil blood counts are shown at baseline and after dupilumab treatment. Blue line in all box plots is connecting median values. BL, baseline; FeNO, fractional exhaled nitric oxide.

A decline of FeNO was observed beginning at the 2–4 months period, showing a reduction of 45.73% [Means: 63.57 (SD 56.69) ppb at BL and 34.50 (23.76) ppb at 2–4 months]. This trend persisted, with mean FeNO levels consistently remaining below 25 ppb after the 5–7 months period (Supplementary Table S5 and Figure 3). To allow for the maximum number of FeNO data points after dupilumab therapy, the last FeNO measurement available for each patient was identified and proportions of patients below the threshold to consider airway inflammation were compared with baseline. Among a total of 47 follow-up data points, 35 patients had FeNO values below 25 ppb (74.5%), which is significantly higher compared to baseline (15 out of 43, 34.9%; chi^2^ = 12.69, *p* = 0.0004).

### 3.7 Blood eosinophil count (BEC)

Median BEC at baseline was 350.0 (IQR: 165.0–635.0) cells/µl; 48 (76.2%) and 37(58.7%) out of 63 (58.7%) patients had counts >150 and >300 cells/µl, respectively. Similar median values were observed during dupilumab treatment, except at 14–18 and 19–25-months periods with median values of 580.00 (IQR: 320–755) and 1,010 (IQR: 505–1,993) cells/µl, respectively (Supplementary Table S5 and Figure 3). Ten patients had BEC above 1,500 cells/µl during dupilumab treatment; however, for none of them eosinophilia was symptomatic. Baseline BEC was available for 7 out of 10 cases (Supplementary Table S6), observing that during dupilumab administration all of them had greater BEC than baseline.

### 3.8 Clinical asthma remission

Only one individual had data from two follow-up time points separated by a year, which enabled the assessment of clinical remission status. Although this individual was in clinical remission, the absence of similar data for the remaining patients made it difficult to determine the proportion of patients who achieved clinical remission within the entire cohort.

### 3.9 Safety

Besides the number of patients within the analyzed cohort used to report clinical outcomes related with asthma control, we considered an additional case to describe the presentation of adverse effects: a patient who discontinued dupilumab at 2 months due to AE; she was 59-years old woman who experienced headache, nervousness, and tremor and discontinued dupilumab. Headache was the most common AE (4.0%, *n* = 4 of 99 patients) followed by conjunctivitis (3.0%, *n* = 3), and sinusitis (3.0%, *n* = 3) (Table 4). Additionally, among the other reported adverse events (Supplementary Table S7), gastrointestinal symptoms (3.0%, *n* = 3), arthralgias (2.0%, *n* = 2), and blurred vision (2.0%, *n* = 2) were the most reported.

**Table 4:** Reported adverse events per person.

Symptom	*N* = 99 (*n*,%)^a^
Headache	4 (4.0%)
Conjunctivitis	3 (3.0%)
Sinusitis	3 (3.0%)
Swelling	1 (1.0%)
Dry eye	1 (1.0%)
Other	13 (13.1%)

^a^ Number of patients with reports of common adverse events.

### 3.10 Temporary discontinuation and complete withdrawal

Twenty patients (20.4%) had temporary discontinuation of dupilumab (mean time: 2.2 months) and some had more than one treatment suspension for a total of 23 events (Supplementary Table S8). Most suspensions (65.2%, *n* = 15) were due to delays in drug administration by the Health Insurance Provider (Supplementary Table S9).

Three (3.1%) patients completely withdrew from the medication after 6 months of usage (with an average duration of 6.3 months for those on dupilumab). The reason for complete withdrawal was not recorded in the medical record in one patient, while two patients withdrew due to adverse events: one experienced gastrointestinal symptom, while the other experienced both gastrointestinal symptoms and a lack of efficacy, leading to a switch to benralizumab.

## 4 Discussion

In this RW study, we documented clinical improvement after dupilumab use in patients with SA treated in different centers in Colombia. We observed significant reductions in the occurrence of severe exacerbations that led to HCRU, and in the proportion of uncontrolled asthma symptoms as reported by ACT. Also, the FeNO decline supported a marked reduction in the inflammatory airway process in most treated patients. In general, the biologic was well-tolerated, observing that adverse events were reported in a similar rate to those reported in pivotal clinical trials (10–12). Although, high BEC was observed in some patients, this was not associated with clinical symptoms that could raise concerns about dupilumab safety.

Exacerbation reduction is an important criterion to assess the effectiveness of an asthma intervention. In this cohort, a reduced severe AER was observed after dupilumab initiation. AER changed from 0.6 to 0.1 and 0.08 in the first and second year of treatment, respectively. This reduction rate of approximately 80%, was notably higher than that reported in other real-world studies from the US (14) and Japan (13), where the reduction was close to 40%, as well as that reported in RCTs (9). A higher reduction in exacerbation rates was published by Caminati et al. in an Italian cohort, wherein the exacerbation numbers decreased from 2.6 to 0.1 at 12 months of therapy (15). Our cohort is more similar to the one described in that study in terms of the number of patients with nasal polyps co-morbidity (15). Prescription of OCS for asthma control was also reduced in this cohort after dupilumab initiation coinciding with other real-word reports (13, 16) and clinical studies (17). The sparing effect on OCS use may have beneficial effects on patients due to well-known adverse events associated with them (18).

Eosinophilia has been reported as an adverse event associated with the use of dupilumab, with cases ranging from asymptomatic to severe (19). Hypereosinophilia at any moment during dupilumab treatment was observed in ∼10% of patients, although this rate varies compared to other studies. However, it is noteworthy that no cases were associated with symptoms. Notably, none of these cases were symptomatic. However, there is a safety concern due to instances of conjunctivitis and gastroenteritis potentially linked to eosinophilia. One proposed mechanism for the increase in eosinophils is that dupilumab's inhibition of the IL-4/IL-13 pathway reduces eosinophil infiltration into inflamed tissue, thereby potentially increasing circulating eosinophils (20). Follow-up studies, such as TRAVERSE, suggest that this hypereosinophilia may be transient. In the TRAVERSE study, which observed patients for 96 weeks, the rise in eosinophils declined over time, with no cases observed after two years. In our cohort, most cases of elevated eosinophil absolute counts (BEC >1,500 cells/ul) were observed during the 22–24-month follow-up period. In contrast, other studies have observed high BEC levels within the first three months, whereas in our study, the median BEC at that time point was similar to baseline levels. Unfortunately, BEC data were unavailable at all time points or at baseline, limiting our ability to draw definitive conclusions about BEC patterns in the Colombian population. Thus, this area warrants further exploration, because other environmental exposures, such as helminth infections, may affect blood eosinophils (12). This observation regarding hypereosinophilia, although seen in a small percentage of patients without associated clinical symptoms, underscores the importance of monitoring eosinophil levels during dupilumab treatment.

FeNO has been proposed as a reliable biomarker to evaluate the impact of biologics on airway inflammation. In this RWE, we observed that 74.5% of patients achieved reduction of FeNO below 25 ppb. This agrees with preliminary publications for the VESTIGE trials that reported an achievement of FeNO normalization in 66% of patients with dupilumab compared with 10% of patients receiving placebo. This improvement in airway inflammation was also evidenced by measuring mucus plugging and airway volume through structural studies (21).

Notably, despite delays or temporary discontinuation in the administration of dupilumab, primarily because of health system-related access difficulties, a favorable clinical response to this biologic was observed, as evidenced by both direct medical and patient-reported outcomes. These findings underscore the importance of RW data, which can be highly valuable in clinical decision-making, particularly in underdeveloped countries, where clinical scenarios may differ significantly from the controlled administration of medication observed in RCTs. Local data can also optimize the cost-effectiveness modeling of health interventions for SA (22). For instance, in Colombia, two studies comparing biologics as interventions for SA used RCTs to assess efficacy (22, 23). Our data can enhance the accuracy and applicability of modeling studies in this context.

This study has limitations that should be noted. First, dupilumab was approved in Colombia in 2019, and the observation window overlaps with the period when COVID-19 pandemic lockdowns affected medical follow-up for patients. Additionally, SARS-CoV-2 infection could have influenced lung function. The regularity of medical visits and controls, as well as attendance for lab tests, could also have been negatively impacted by the pandemic. Second, the use of OCS was not considered as part of the criteria to define severe exacerbations due to concerns about patients self-administering OCS regardless of the severity, as well as the lack of reporting in medical records. Since most studies use this criterion to define severe exacerbations, AER could be underestimated in this cohort. Additionally, although the observed reduction in AER after one and two years of treatment could theoretically be subject to attrition bias—if patients with more frequent exacerbations discontinued treatment or follow-up—this risk is likely minimal in our cohort. Only 3 out of 98 patients (3.1%) dropped out during the study period, suggesting that the impact of patient attrition on outcome estimates is limited. Although we attempted to retrieve as much data as possible from clinical records, missing data at various time points limited the ability to perform repeated measures analyses. As a result, we opted for independent group comparisons based on rates at baseline and follow-up time points. Also, it should be noted that no sample size estimation was performed to ensure the robustness of these comparisons. This study did not include data from claims databases that register all prescriptions for a patient as reported for other studies, but only those from the specialized consultation service if reported in the medical record. However, an advantage is that different clinical and biological criteria, used in real practice, could also be analyzed, and support an improvement in asthma symptoms and disease control. This study highlights the importance of generating local data to optimize cost-effectiveness models for health interventions in the treatment of severe asthma in developing countries. Additionally, it can provide a stronger basis for clinical decision-making and health policy formulation.

In conclusion, dupilumab is an effective and well-tolerated treatment for severe asthma in Colombia, showing substantial reductions in severe exacerbations and improvements in asthma control and FeNO values. Ongoing research and long-term follow-up studies are essential to further validate these findings and explore the underlying mechanisms of eosinophilia in treated patients.

## Data Availability

The raw data supporting the conclusions of this article will be made available by the authors, without undue reservation.
