# Supplementary material for: Real world evidence of dupilumab effectiveness in a Colombian cohort of patients diagnosed with severe asthma
Source: Front Allergy. 2025 May 13;6:1564033. doi: 10.3389/falgy.2025.1564033 (PMC12106408; doi:10.3389/falgy.2025.1564033)
Supplement: Supplementary file 1 [file Datasheet1.docx]

Supplementary

Table S1. Mean treatment duration of dupilumab by clinical center

| Clinical care center | Months  Mean (SD) |
| --- | --- |
| Fundación Neumológica Colombiana | 19.33 (5.55) |
| UNIMEQ | 12.11 (5.39) |
| Neumomed IPS | 12.88 (8.13) |
| Instituto Neumológico del Oriente | 9.45 (4.66) |
| Centro de Alergología Alejandro Carreño | 16.25 (7.37) |
| **Total** | **14.39 (7.08)** |

Table S2. Mean annual rates of asthma exacerbation, hospital admission, ER visit and ICU admission.

|  | BL (N=98) | T1 (N=26) | T2 (N=26) | p-value |
| --- | --- | --- | --- | --- |
| Severe exacerbation. Mean (SD) | 0.61 (1.45) | 0.11 (0.54) | 0.08 (0.20) | 0.03 |
| ER visits. Mean (SD) | 0.33 (1.16) | 0.07 (0.36) | 0.06 (0.18) | 0.26 |
| Hospitalization. Mean (SD) | 0.29 (0.79) | 0.04 (0.18) | 0.02 (0.09) | 0.08 |
|  |  |  |  |  |

Table S3. Usage of concomitant medication in the cohort

|  | BL (N=98) | 2-4 (N=72) | 5-7 (N=42) | 8-10 (N=40) | 11-13 (N=26) | 14-18 (N=43) | 19-25 (N=27) |
| --- | --- | --- | --- | --- | --- | --- | --- |
| **OCS** |  |  |  |  |  |  |  |
| No | 78 (79.6%) | 61 (84.7%) | 35 (83.3%) | 39 (97.5%) | 24 (92.3%) | 41 (95.3%) | 25 (92.6%) |
| Yes | 20 (20.4%) | 11 (15.3%) | 7 (16.7%) | 1 (2.5%) | 2 (7.7%) | 2 (4.7%) | 2 (7.4%) |
| **ICS/LABA** |  |  |  |  |  |  |  |
| No | 5 (5.1%) | 3 (4.2%) | 3 (7.1%) | 3 (7.5%) | 2 (7.7%) | 1 (2.3%) | 2 (7.4%) |
| Yes | 93 (94.9%) | 69 (95.8%) | 39 (92.9%) | 37 (92.5%) | 24 (92.3%) | 42 (97.7%) | 25 (92.6%) |
| **ICS** |  |  |  |  |  |  |  |
| No | 92 (93.9%) | 69 (95.8%) | 41 (97.6%) | 39 (97.5%) | 26 (100.0%) | 41 (95.3%) | 25 (92.6%) |
| Yes | 6 (6.1%) | 3 (4.2%) | 1 (2.4%) | 1 (2.5%) | 0 (0.0%) | 2 (4.7%) | 2 (7.4%) |

Table S4. Changes from baseline and total value of Asthma Control Test.

|  | BL (N=75) | 2-4 (N=48) | 5-7 (N=30) | 8-10 (N=22) | 11-13 (N=19) | 14-18 (N=28) | 19-25 (N=19) |
| --- | --- | --- | --- | --- | --- | --- | --- |
| **Change in ACT score** | |  |  |  |  |  |  |
| Mean (SD) | 0 | 4.53 (5.24) | 5.48 (3.55) | 5.39 (4.77) | 6.88 (6.06) | 4.35 (4.63) | 5.25 (4.68) |
| Median (IQR) | 0 | 3.50 (1.00, 8.75) | 6.00 (3.00, 8.00) | 6.00 (4.00, 8.25) | 6.00 (1.75, 11.00) | 4.00 (2.50, 7.25) | 5.00 (1.50, 8.25) |
| **Total value ACT score** | |  |  |  |  |  |  |
| Mean (SD) | 13.91 (4.60) | 17.73 (5.53) | 18.97 (4.55) | 18.91 (5.00) | 20.21 (2.80) | 18.75 (5.34) | 20.63 (3.45) |
| Median (IQR) | 13.00 (11.00, 17.00) | 20.00 (13.00, 22.25) | 20.00 (16.25, 22.75) | 20.50 (15.25, 23.00) | 21.00 (19.50, 22.00) | 20.00 (15.75, 23.25) | 22.00 (19.00, 23.00) |

Table S5. Changes from baseline in FeNO, eosinophil blood counts and IgE values

|  | BL | 2-4 | 5-7 | 8-10 | 11-13 | 14-18 | 19-25 |
| --- | --- | --- | --- | --- | --- | --- | --- |
| **FeNO** |  |  |  |  |  |  |  |
| Total | 42 | 10 | 16 | 7 | 10 | 10 | 5 |
| Mean (SD) | 63.57 (56.69) | 34.50 (23.76) | 22.75 (21.97) | 25.00 (19.88) | 16.50 (9.32) | 21.50 (22.70) | 38.00 (24.62) |
| Median (IQR) | 47.00 (20.50, 96.00) | 33.00 (17.50, 48.25) | 14.50 (9.75, 25.50) | 16.00 (13.50, 32.50) | 18.50 (8.75, 22.75) | 15.00 (11.50, 16.75) | 31.00 (28.00, 43.00) |
| **Eosinophils (cells/uL)** | | |  |  |  |  |  |
| Total | 63 | 16 | 13 | 11 | 9 | 11 | 7 |
| Mean (SD) | 513.79 (659.95) | 618.38 (657.45) | 368.08 (320.56) | 894.27 (1194.34) | 563.89 (598.41) | 669.91 (522.14) | 1426.29 (1403.67) |
| Median (IQR) | 350.00 (165.00, 635.00) | 340.00 (192.50, 895.00) | 320.00 (153.00, 412.00) | 560.00 (190.00, 1095.00) | 250.00 (220.00, 440.00) | 580.00 (320.00, 754.50) | 1010.00 (505.00, 1993.00) |
| **IgE (kU/L)** | |  |  |  |  |  |  |
| Total | 54 | 7 | 6 | 3 | 4 | 1 | 1 |
| Mean (SD) | 544.74 (776.66) | 291.43 (430.49) | 175.50 (294.43) | 286.67 (308.28) | 27.00 (24.39) | 72.00 (NA) | 45.00 (NA) |
| Median (IQR) | 183.50 (52.00, 685.00) | 129.00 (81.50, 242.00) | 64.50 (14.75, 136.75) | 176.00 (112.50, 405.50) | 22.00 (16.50, 32.50) | 72.00 (72.00, 72.00) | 45.00 (45.00, 45.00) |

Table S6. Baseline blood eosinophil absolute counts in patients with eosinophilia (1500 cells/uL) after dubilumab initiation.

| **ID** | **Baseline** | **Control** |
| --- | --- | --- |
| 27 | N.A. | 7120 |
| 16 | 70 | 4240 |
| 31 | 479 | 3890 |
| 02 | N.A. | 2836 |
| 07 | 130 | 2270 |
| 19 | 480 | 1820 |
| 11 | 380 | 1730 |
| 07 | 130 | 1600 |
| 17 | 1390 | 1590 |
| 30 | N.A. | 1550 |

N.A. Not available

Table S7. Other adverse events

| Symptoms | N =99  (n,%)* |
| --- | --- |
| Gastrointestinal | 3 (3.0%) |
| Arthralgias | 2 (2.0%) |
| Blurred vision | 2 (2.0%) |
| Diplopia | 1 (1.0%) |
| Edema in the feet, hands and face | 1 (1.0%) |
| Vertigo | 1 (1.0%) |
| Dizziness and emesis | 1 (1.0%) |
| Pruritic lesions on the chest | 1 (1.0%) |
| Sleep impairment | 1 (1.0%) |
| Myalgias | 1 (1.0%) |
| Cough | 1 (1.0%) |
| Ocular pruritus | 1 (1.0%) |
| Self-limiting chest pain | 1 (1.0%) |
| Angioedema in the mouth and eyelids and generalized pruritus | 1 (1.0%) |
| Nervousness | 1 (1.0%) |
| Tremor | 1 (1.0%) |

*Number of patients with other reports of adverse events.

Table S8. Monthly Frequency of Temporary Discontinuation of Dupilumab

| Months | N (%) |
| --- | --- |
| 1 | 11 (47.8%) |
| 2 | 8 (34.8%) |
| 4 | 1 (4.3%) |
| 5 | 1 (4.3%) |
| 6 | 1 (4.3%) |
| 9 | 1 (4.3%) |
| Mean (SD) | 2.2 (2.0) |
| Total | 23 |

Table S9. Reasons for temporary discontinuation

| **Reason** | **N (%)** |
| --- | --- |
| Lack of authorization | 15 (65.2%) |
| Personal problems | 5 (21.7%) |
| Adverse event | 0 (0.0%) |
| Medication availability | 3 (13.0%) |
| Other | 9 (39.1%) |
